# Supplementary figures and images for: Rev-RRE activity modulates HIV-1 replication and latency reactivation: Implications for viral persistence and cure strategies
Source: PLoS Pathog. 2025 May 15;21(5):e1012885. doi: 10.1371/journal.ppat.1012885 (PMC12080775; doi:10.1371/journal.ppat.1012885)

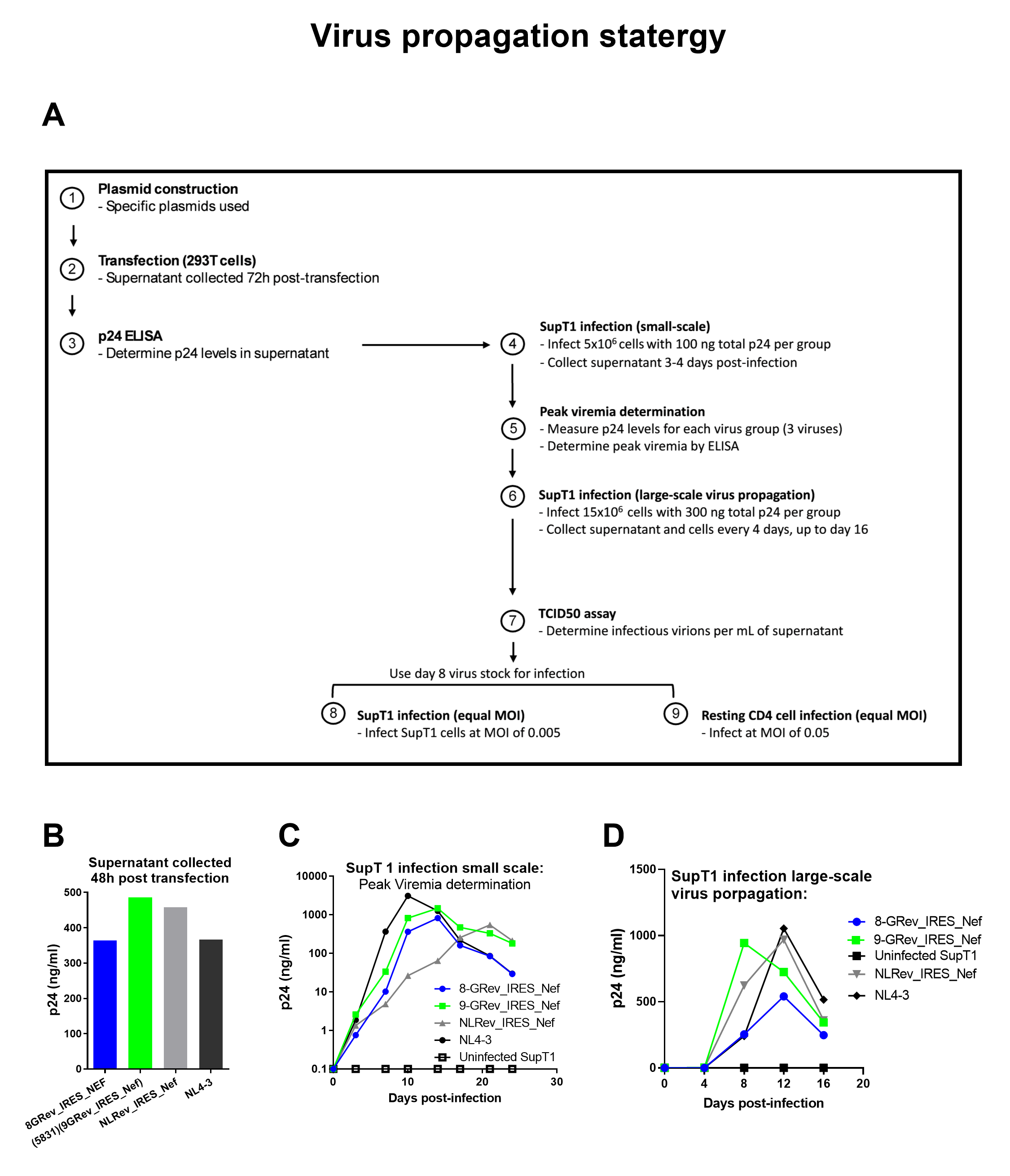

Supplement: S1 Fig — (A) Schematic representation of the virus propagation strategy. (B) 293T cells were transfected with pNLRev_IRES_Nef (HamRek #5833), p8-GRev_IRES_Nef (HamRek #5830), p9-GRev-IRES_Nef (HamRek #5831), and original pNL4–3 plasmids. P24 levels in supernatants were measured 48 hours post-transfection. (C) Initial infection kinetics in SupT1 cells (1x10^6^ cells/ml) using 100 ng p24 from transfection supernatants. Peak viremia timepoints were selected for large stock production. (D) Infection kinetics in SupT1 cells using peak p24 virus stock from (C) to generate final expanded viral stocks. TCID50 was determined for these stocks. Day 8 stock, which showed the highest titer, was used for infection in subsequent experiments. (TIF) [file ppat.1012885.s001.tif]

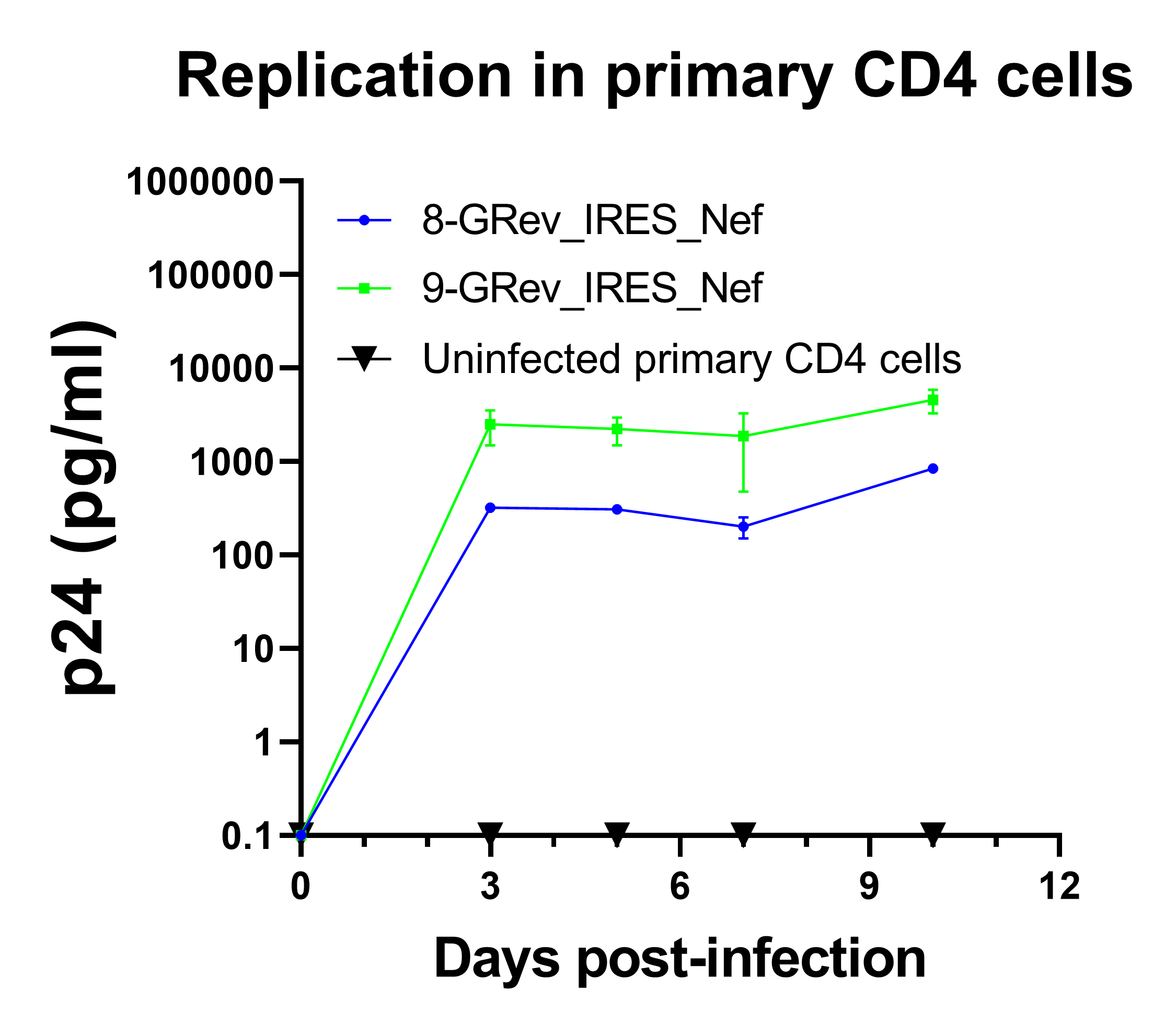

Supplement: S2 Fig — Resting CD4 ⁺ T cells were pre-activated for 48 hours with PHA (10 µg/mL) plus IL-2 (10 IU/mL) and then infected at an MOI of 0.05 by spinoculation for 1 hour at room temperature. Cells were washed three times with room temperature 1X DPBS to remove unbound virus, cultured in RPMI supplemented with 10% FBS and IL-2, and supernatants were collected every 3–4 days for p24 quantification. Plotted p24 values represent corrected concentrations that account for the residual 50 µ L of supernatant left behind after each media change. The 9-GRev_IRES_Nef virus (green) consistently replicated more efficiently than 8-GRev_IRES_Nef (blue), consistent with observations in SupT1 cells. Data are mean ± SEM from triplicate experiments. (TIF) [file ppat.1012885.s002.tif]

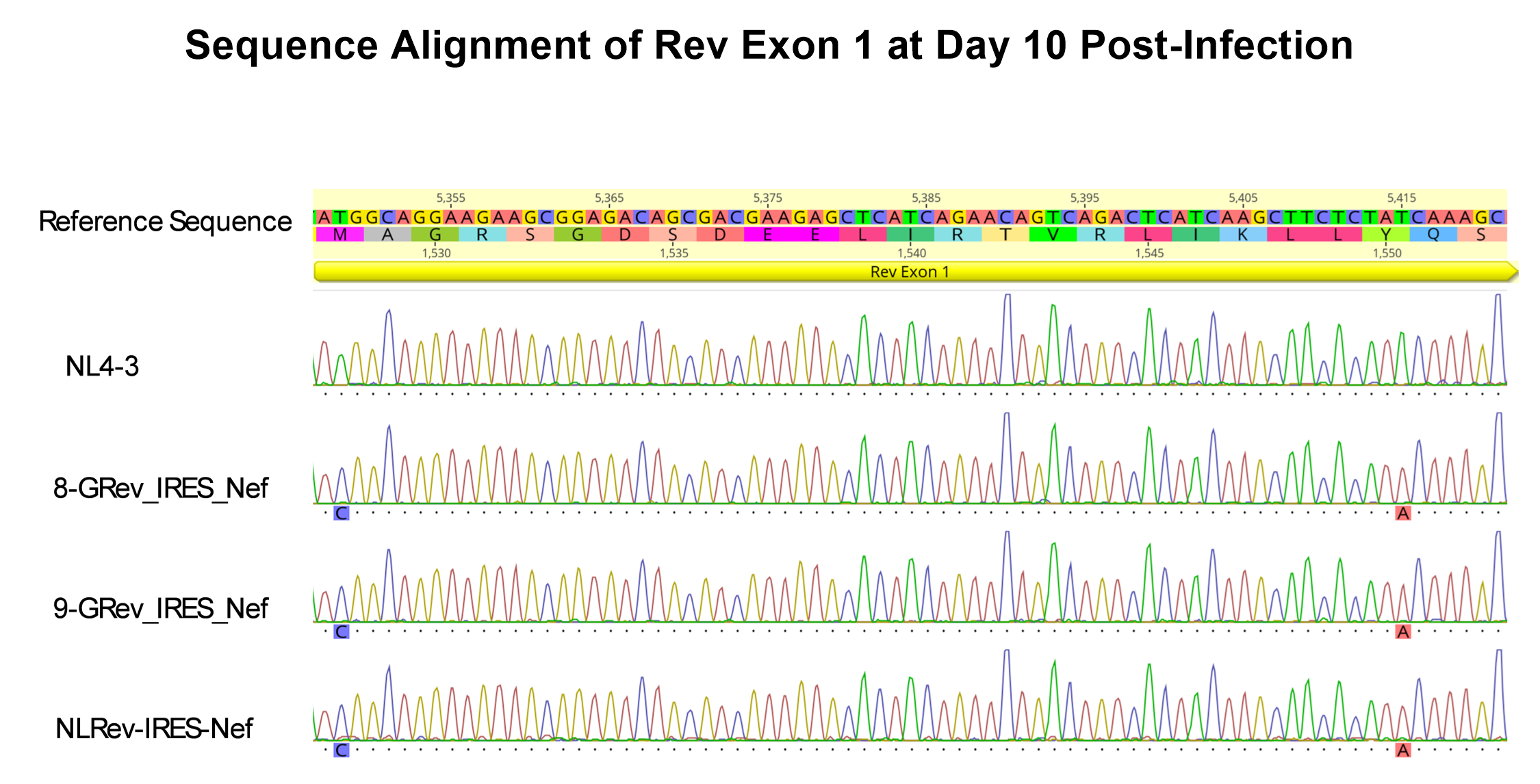

Supplement: S3 Fig — Late-timepoint (day 10) infected SupT1 cells were harvested, and total genomic DNA was subjected to PCR and Sanger sequencing. The sequencing chromatograms show that neither the introduced stop codon (UAA, replacing the native UAU) nor the disrupted start codon (ACG, replacing AUG) in exon 1 of rev underwent reversion during viral replication. These findings confirm that the relocated Rev-IRES-Nef cassette remained intact and that no selective advantage for revertants was observed under our experimental conditions. (TIF) [file ppat.1012885.s003.tif]

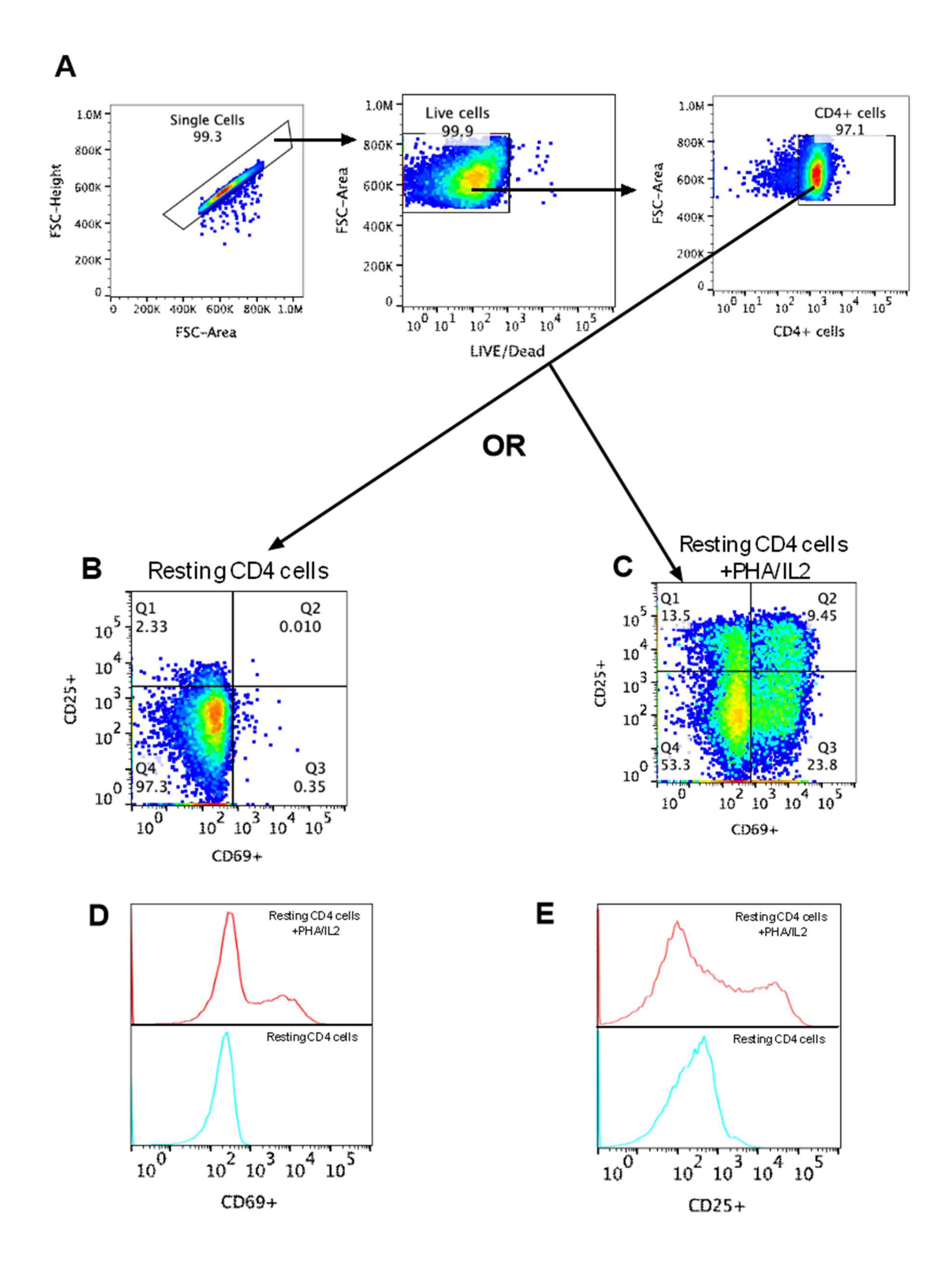

Supplement: S4 Fig — (A) The Gating strategy used to identify live CD4 + T cells from total events: live cells were first selected, followed by gating on CD4 ⁺ singlets. (B) Representative dot plot showing minimal CD69 and CD25 expression in CCL19-treated, resting CD4 + T cells. (C) Corresponding dot plot of activated CD4 ⁺ T cells treated with phytohaemagglutinin (PHA) plus interleukin-2 (IL-2) for 24 hours, demonstrating robust upregulation of both CD69 and CD25. (D) Histograms comparing CD69 expression levels in resting (blue) versus activated (red) cells. (E) Histograms comparing CD25 expression in resting (blue) versus activated (red) cells. Taken together, these data confirm that CCL19 maintains cells in a quiescent state, whereas PHA/IL-2 triggers a high-activation phenotype. (TIF) [file ppat.1012885.s004.tif]

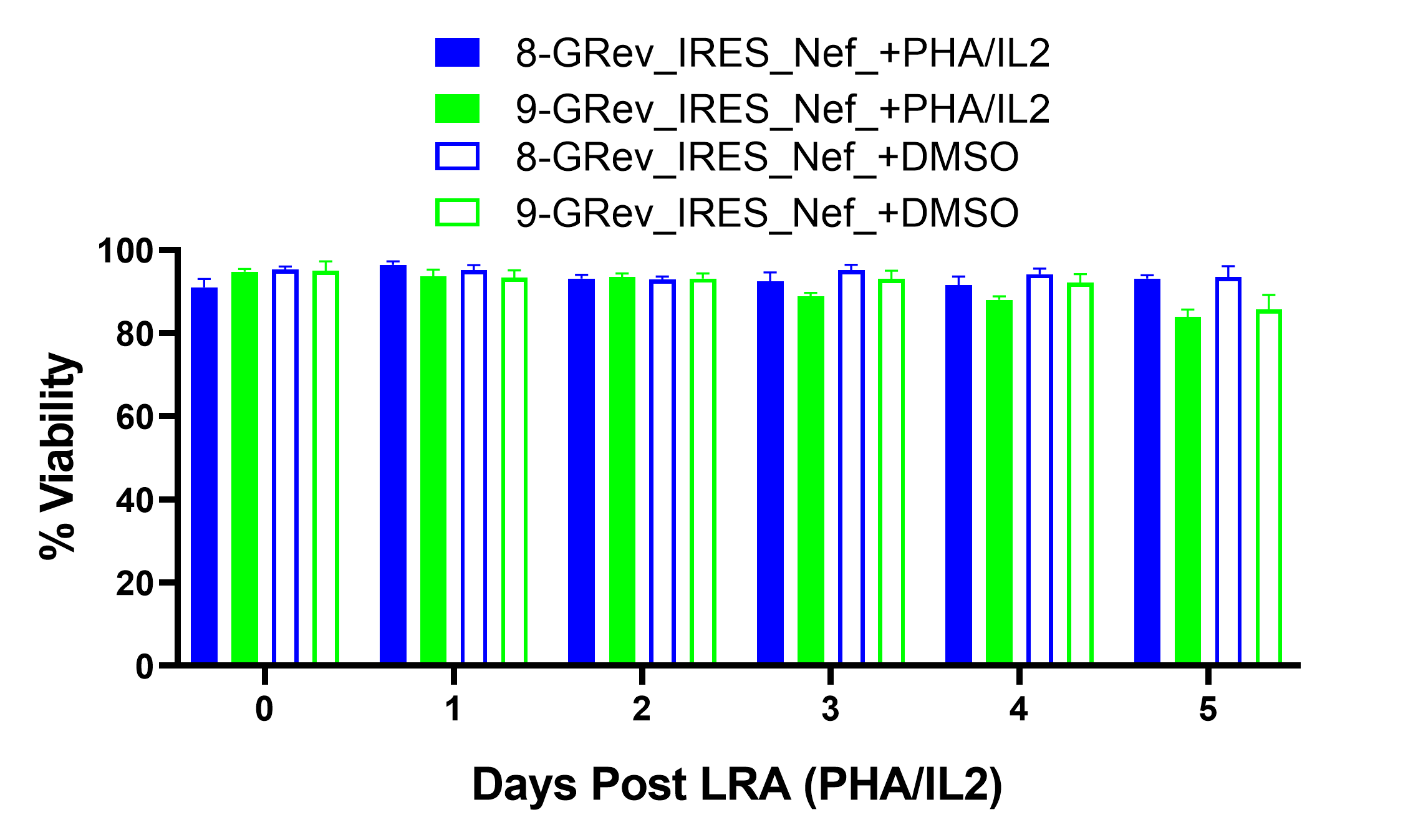

Supplement: S5 Fig — Cell viability of resting CD4 + T cells latently infected with 8-GRev_IRES_Nef or 9-GRev_IRES_Nef viruses, treated with latency reversing agents (LRA) or DMSO vehicle at day 3 post-infection, and cultured in the presence of zidovudine (AZT) to prevent reinfection. Cell viability was assessed using trypan blue staining from day 1–5 post-reactivation. The graph shows the percentage of viable cells over time for both viral constructs under LRA and AZT treatment conditions. (TIF) [file ppat.1012885.s005.tif]

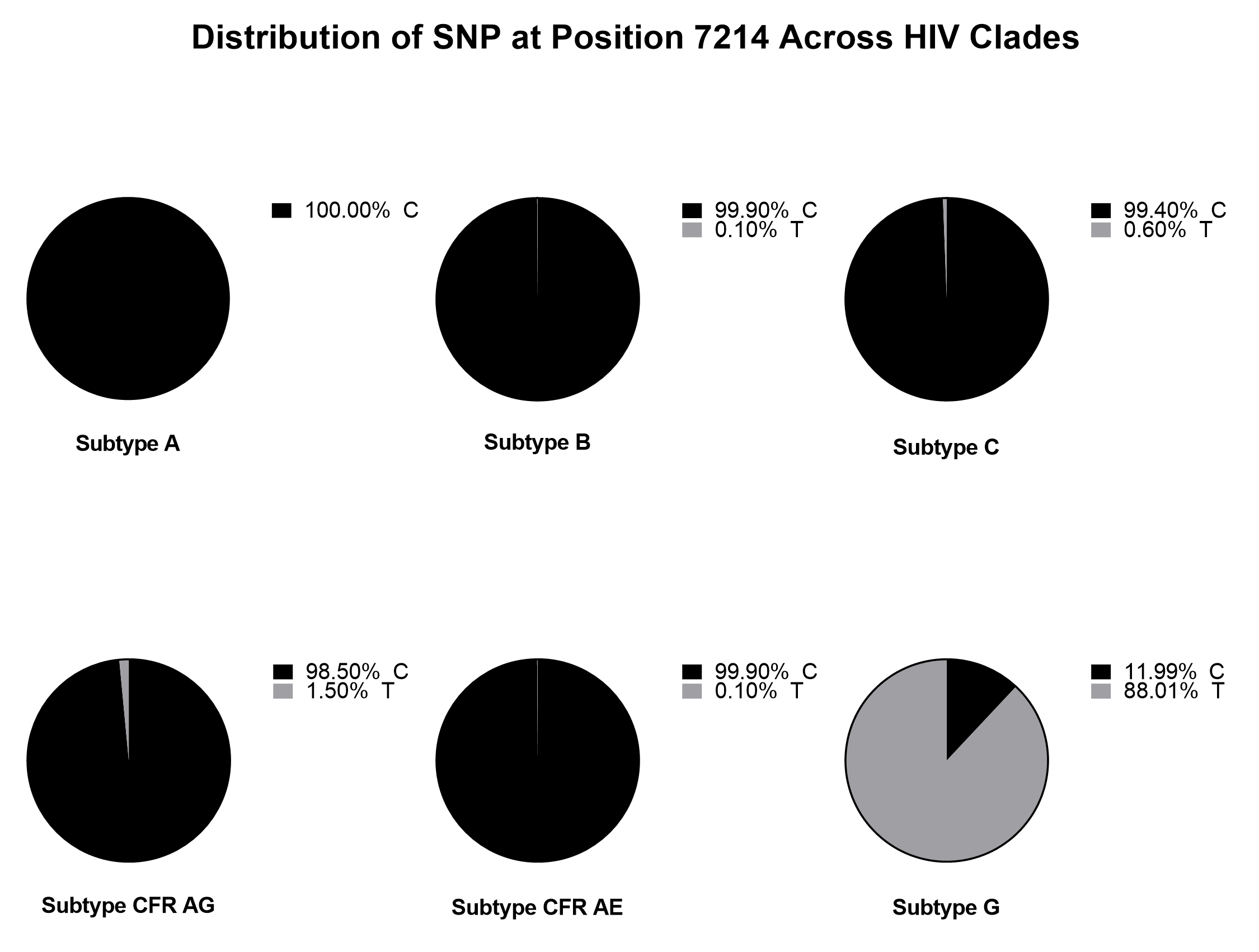

Supplement: S6 Fig — To assess whether C7214T is commonly present in naturally circulating HIV-1 strains, we surveyed RRE sequences from multiple subtypes in the Los Alamos HIV Database, as described in Materials and Methods. Each pie chart depicts the proportion of sequences harboring thymidine (T) versus cytidine (C) at position 7214 (relative to NL4–3 numbering) among major HIV-1 subtypes: A (n = 96), B (n = 70,689), C (n = 29,733), G (n = 603), CRF_AE (n = 10,059), and CRF_AG (n = 1,242). (TIF) [file ppat.1012885.s006.tif]
